# Species-specific duplications of NBS-encoding genes in Chinese chestnut (*Castanea mollissima*)

**DOI:** 10.1038/srep16638

**Published:** 2015-11-12

**Authors:** Yan Zhong, Yingjun Li, Kaihui Huang, Zong-Ming Cheng

**Affiliations:** 1 College of Horticulture, Nanjing Agricultural University, Nanjing 210095, China

## Abstract

The disease resistance (*R*) genes play an important role in protecting plants from infection by diverse pathogens in the environment. The nucleotide-binding site (NBS)-leucine-rich repeat (LRR) class of genes is one of the largest *R* gene families. Chinese chestnut (*Castanea mollissima*) is resistant to Chestnut Blight Disease, but relatively little is known about the resistance mechanism. We identified 519 NBS-encoding genes, including 374 NBS-LRR genes and 145 NBS-only genes. The majority of *Ka/Ks* were less than 1, suggesting the purifying selection operated during the evolutionary history of NBS-encoding genes. A minority (4/34) of *Ka/Ks* in non-TIR gene families were greater than 1, showing that some genes were under positive selection pressure. Furthermore, *Ks* peaked at a range of 0.4 to 0.5, indicating that ancient duplications arose during the evolution. The relationship between *Ka/Ks* and *Ks* indicated greater selective pressure on the newer and older genes with the critical value of *Ks* *=* 0.4–0.5. Notably, species-specific duplications were detected in NBS-encoding genes. In addition, the group of RPW8-NBS-encoding genes clustered together as an independent clade located at a relatively basal position in the phylogenetic tree. Many *cis*-acting elements related to plant defense responses were detected in promoters of NBS-encoding genes.

Plants have a large number of disease RESISTANCE (*R*) genes for defense against numerous and various pathogens, including bacteria, fungi, oomycetes, viruses, and nematodes^1^,^2^,^3^. *R* genes encode proteins that allow plants to systematically recognize and respond to pathogen infection^4^,^5^,^6^. NBS-LRR genes are one of the largest gene families in plant genomes and the largest class of known disease resistance genes. Among the over 140 *R* genes characterized from different flowering plants, approximately 80% belong to the NBS-LRR (nucleotide-binding site and leucine-rich repeat) gene families, members of which can directly or indirectly identify the pathogen^2^,^7^,^8^.

The NBS domain is thought to be required in binding and hydrolyzing ATP and GTP^1^. The LRR motif in NBS-encoding genes functions is to regulate direct or indirect interactions with pathogens^9^. Based on the presence or absence of an N-terminal Toll/Interleukin-1 receptor (TIR) domain, NBS-encoding genes are divided into TIR-NBS-LRR/TIR-NBS (TNL/TN) genes and non-TIR-NBS-LRR/non-TIR-NBS (non-TNL/non-TN) genes^10^. The latter usually has a coiled-coil (CC) or other domains at the N-terminus, therefore this category can be further divided into CC-NBS-LRR/CC-NBS genes (CNL/CN) and X-NBS-LRR/X-NBS genes (XNL/XN)^1^. CC and LRR domains co-regulate the signaling capacity of the NBS domain in a recognition-specific manner^11^. Further, some NBS-encoding genes have another domain RPW8, which confers resistance to powdery mildew with a transmembrane region before the CC structure at N-terminus^12^. The RPW8-NBS-LRR (RNL) group is generally regarded as a small but special subclass of non-TNL^13^,^14^. However, recent studies indicate that the RNL group is an individual sister group to the non-TNL group^15^,^16^,^17^.

Gene duplications have contributed to the high numbers and proportions of NBS-LRR genes in plant families^18^. The 174, 519, 416 NBS-LRR genes have been identified in *Arabidopsis,* rice, and poplar, the model systems for dicots, monocots, and woody plants, respectively^2^,^19^,^20^,^21^. Chestnut acts as a model species for the Fagaceae that dominate the hardwood forests of the northern hemisphere^22^ and have significant economic and ecological value. Chestnut can be infected with pathogens that cause diseases such as chestnut blight^23^,^24^, ink disease^25^ or bark disease^26^,^27^. Furthermore, many disease resistance genes belonging to the NBS-LRR gene families have been identified^9^,^28^,^29^,^30^,^31^,^32^,^33^,^34^,^35^.

Chestnuts (*Castanea spp.*) are important nuts and forest trees, and play important roles in ecosystem and generate nuts for wildlife and specialty nuts for human consumption. Chinese chestnut is resistant to chestnut blight, caused by fungal pathogen *Cryphonectria parasitica* (formerly *Endothia parasitica*), which, when it was accidentally introduced to North America around 1904 from Japan with nursery stocks, has almost wiped out the American chestnut tree (*Castanea dentata*) in the early 1900’s, once plentiful tree in the eastern United States. However, the resistance mechanism to chestnut blight in Chinese chestnut is not clear. The whole genome sequence recently released^22^,^36^ provides the opportunity to undertake a whole-genome analysis of NBS-encoding *R* genes in Chinese chestnut to obtain insight into the evolutionary development of this gene family. We used poplar (*Populus tricocarpa*)^21^,^37^ as a reference, which was the most closely related to chestnut among all the sequenced species, to identify chestnut-specific duplications after the divergence between Chinese chestnut and poplar. Results of this genome-wide analysis suggests that ancient and species-specific duplications have contributed to the expansion of NBS-encoding genes in Chinese chestnut. This research lays a foundation for further characterizing these *R* genes and helps identifying *R* genes that may be involved in resistance to chestnut blight.

## Results

### Total number of NBS-encoding genes in Chinese chestnut

A total of 519 NBS-encoding genes were identified in the *C. mollissima* genome, including 374 NBS-LRR genes and 145 NBS-only genes (Table 1). The NBS-encoding genes comprised 1.36% of expressed genes in Chinese chestnut compared with 0.91% in poplar. Accordingly, the proportion of NBS-LRR genes in Chinese chestnut (0.98%) was also higher than that in poplar (0.72%). Among NBS-LRR and NBS-only genes, two types of genes could be subclassified based on their N-terminal structures: TIRs and non-TIRs. Among the NBS-encoding genes, 27 TIR genes (22 TNLs and 5 TNs) and 492 non-TIR genes (352 non-TNLs and 140 non-TNs) were found. These results demonstrated that the number of non-TIR genes was greater than that of TIR genes, which was similar to the numbers of the two types of NBS-encoding genes in poplar. Furthermore, the proportions of non-TNLs were greater than these of TNLs in both Chinese chestnut and poplar genomes. In the Chinese chestnut genome, 0.06% and 0.92% of genes detected were TNLs and non-TNLs, respectively, compared to proportions of 0.17% and 0.55% for those gene categories in poplar. However, although the number of NBS-encoding genes in Chinese chestnut was greater than that in poplar, the number of TIR genes in Chinese chestnut was smaller than that in poplar. Moreover, the non-TIR genes could also be further divided into 32 CNLs, 96 CNs, 320 XNLs, and 44 XNs (Table 1).

The average number of exons identified in NBS-LRR genes from Chinese chestnut was 4.37, which was greater than the average of 2.35 exons for NBS-LRR genes in poplar. In addition, the average number of exon in TNL genes was less than that in non-TNL genes in Chinese chestnut, which was different from the average exon numbers in these genes in grape and poplar^21^. The average exon number in TNL, non-TNL, CNL, NBS-encoding, and NBS-LRR genes was 2.50, 2.93, 3.32, 2.66, and 2.90, respectively, which were all less than for all genes predicted in the Chinese chestnut genome.

### Duplication of NBS-encoding genes in the Chinese chestnut genome

According to criteria for both coverage ≥70% and identity ≥70%, 273 genes were detected in 64 NBS gene families in Chinese chestnut. Therefore, 246 of these genes were singletons in the Chinese chestnut genome (Table 2). The percentage of multiple NBS-encoding genes in Chinese chestnut (52.60%) was much lower than that in poplar (78.13%). The average number of members per family was 4.27 in chestnut and 5.33 in poplar, and the maximal number members within a family of these genes in Chinese chestnut (19) was less than that in poplar (23), which indicated fewer NBS gene duplications and multi-gene families in Chinese chestnut than in poplar.

If coverage and identity criteria were changed to 80%, the proportion of multiple genes among all NBS-encoding genes decreased in both Chinese chestnut and poplar. The proportions of multiple NBS-encoding genes were 41.43% and 74.52% between Chinese chestnut and poplar, respectively, which could allow the inference that more relatively recent duplications have occurred in the poplar genome. When coverage and identity criteria were increased to 90%, 19.46% of the multiple genes were still identified in Chinese chestnut, indicating that recent duplication events partly contributed to the expansion of NBS-encoding genes.

The multi-gene families and number of multiple genes encoding TNLs and non-TNLs were diverse in Chinese chestnut. The numbers of both multi-gene families and multiple genes in non-TNLs were greater than those in TNLs, indicating that duplication of NBS-encoding genes occurred primarily among non-TNL genes (Table 2).

### Duplication time of NBS-LRR genes in chestnut

**Figure 1:**
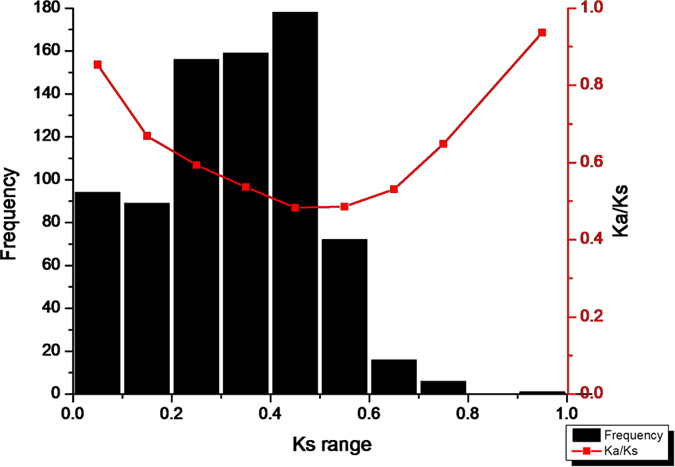
The frequency distribution of relative *Ks* nodes (bar chart) and the relationship between *Ks* and *Ka/Ks* (line chart). The *X-*axis denotes average *Ks* per unit of 0.1 and *Y-*axis denotes frequency and average *Ka/Ks* ratios, respectively.

*Ks* is the time indicator for duplication events, and the frequency distributions of individual *Ks* values reflect the relative time of genome duplications^21^. Firstly, we calculated the rate of synonymous substitutions and obtained the frequency distribution of *Ks* values in non-TIR-NBS-encoding genes (Fig. 1), which had a significant *Ks* peak in the range from 0.4 to 0.5. However the tendency decreased when *Ks* values ranged from 0.5 to 1. *Ks* values in the range from 0.1 to 0.2 accounted for 12.19% of the values in the range of *Ks* values between 0 and 1.0, which demonstrated relatively recent duplications in Chinese chestnut. However, the *Ks* range from 0.4 to 1.0 indicates that 35.41% of the duplication events occurred during chronologically relatively distant periods. Secondly, the curve between *Ka/Ks* and *Ks* for non-TIR-NBS-encoding genes in Chinese chestnut was fitted to detect any relationship between evolutionary pressure and duplication time for NBS paralogs. It was clear that the younger and older genes had larger *Ka/Ks* values, indicating that they were under greater selective pressures within the critical range of *Ks* values between 0.4 and 0.5.

However, among TIR-NBS-encoding genes, only two *Ks* values (0.4781 and 0.1624) were calculated from two gene families including four members, with *Ka/Ks* values of 0.3606 and 0.4554, respectively, which were lower than the average *Ka/Ks* value for non-TIR gene families, indicating greater functional constraints in TIR gene families.

### Selective pressure on NBS-encoding genes in Chinese chestnut

Plant disease resistance genes have been shown to be subject to positive selection^38^. To better understand the evolutionary fate of NBS-encoding duplicates in Chinese chestnut, we used site and branch models in PAML4 to detect positive selection patterns. Because this analysis requires comparison of at least three genes, TIR-NBS-encoding genes were not evaluated and 34 non-TIR genes families with greater than three members each were analyzed.

**Figure 2:**
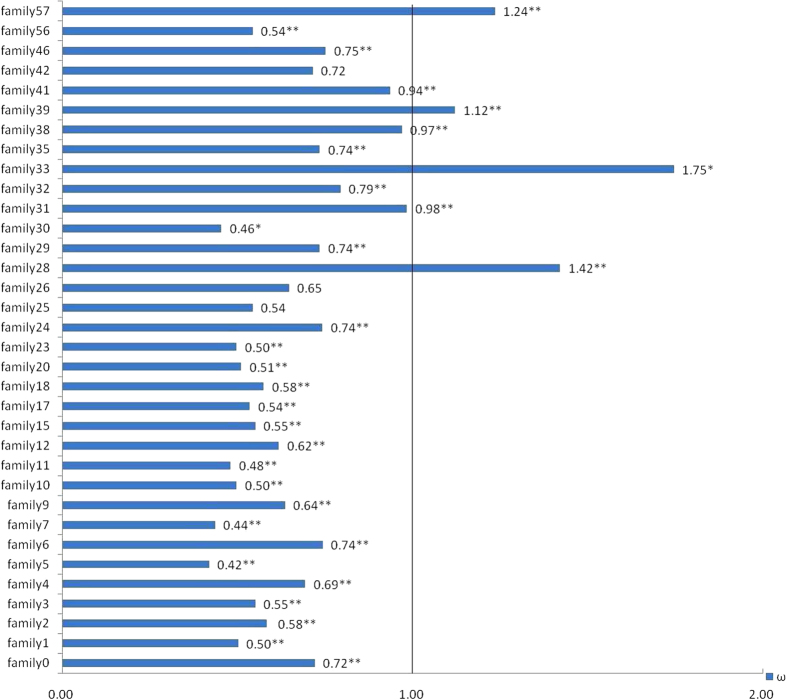
Selective pressure on non-TIR-NBS-encoding genes in Chinese chestnut. Numbers represent the *dN/dS* ratio for each gene family using the branch model; 2∆ln represents the result of the LR test for the site model; * and ** represent, respectively, significant (2∆ln > 5.991, *p* < 0.05) and highly significant (2∆ln > 9.210, *p* < 0.01) tests for positive selection between model M7 and M8.

We calculated the ratios of nonsynonymous to synonymous nucleotide substitutions (ω), a molecular evolutionary measure of selection pressure^39^, to detect positive selection in NBS-encoding genes. A value of ω greater than 1 indicates that a gene is evolving with more constraint on nonsynonymous substitutions than on synonymous substitutions, which is evidence of positive selection^40^. In contrast, value of ω less than 1 indicate purifying selection. A value for ω of 1 means that a gene is under neutral selection. As Fig. 2 shows, most of these gene families (30/34) have undergone purifying selection. Meanwhile, four gene families had values for ω of greater than 1, which demonstrated that some NBS-encoding gene families were under positive selection.

Subsequently, the result of the LR test, 2Δln detected positive selection of significant differences between the M7 and M8. It was important to note that 85.29% (29/34) (Fig. 2) of non-TIR gene families had some sites under highly significant positive selection pressure, showing positive selection on gene families played a certain role in the evolution of non-TIR-NBS-encoding genes. Specifically, the analysis revealed that two gene families (Family 30 and 33) (Fig. 2) were driven by significant positive selection. Further, Bayes Empirical Bayes (BEB) analysis was performed to detect amino acid sites that have been under positive selection^41^. The sites inferred to be under positive selection at the 95% (*) and 99% (**) confidence intervals are shown in Table S1 (Additional file 1), which suggested that these positively selected sites possessed relatively high substitutions compared with others among NBS-LRR genes.

Taken together, 530 amino acid sites from 22 families were revealed to be under positive selection in non-TIR gene families, which might have driven the evolution of function in NBS-encoding genes in Chinese chestnut (Additional file 1: Table S1).

### Phylogenetic analysis of NBS-encoding genes

To confirm the phylogenetic relationships among the NBS-encoding genes, the NBS domains of 519 NBS-encoding genes were analyzed and compared with those in poplar, and a phylogenetic tree was constructed. A few genes had longer branch lengths but the majority had relatively shorter branches (Additional file 2: Figure S1), which indicated two different evolutionary patterns occurred in NBS-encoding genes.

In general, the majority of the NBS-encoding genes were clustered according to species, which suggested species-specific duplication during the evolution of Chinese chestnut. In the phylogenetic tree, when the clade was defined by bootstrap values of greater than 50%, the clades resulting from species-specific duplication were counted. Specifically, 81 clades, including 401 NBS-encoding genes were identified with 4.95 *R* paralogs per clade. Additionally, the proportion of species-specific duplicated genes in Chinese chestnut was 77.26%. This result clearly indicated that NBS-encoding genes expanded into multiple gene family members after the divergence of Chinese chestnut and poplar.

The N-terminal RPW8 domain (RESISTANCE TO POWDERY MILDEW8) in the NBS-encoding genes, medicates broad-spectrum resistance in *Arabidopsis*^12^. The analysis of RPW8 genes helps explain the origin and relationships of RPW8 genes to other genes. In the present study, a total of 15 Chinese chestnut RPW8-NBS-encoding genes are marked with solid circle in phylogenetic tree (Additional file 2: Figure S1). For comparison, five NBS-encoding genes from poplar containing the RPW8 domain are marked with triangle in poplar (Additional file 2: Figure S1). Interestingly, the genes carrying the RPW8 domain were clustered together (Additional file 2: Figure S1).

Additionally, the 15 RPW8 genes from Chinese chestnut formed a relatively independent and monophyletic group, that was not phylogenetically embedded within other clades. Similar results were obtained for RNL genes from *M. truncatula*, potato, soybean, common bean and pigeon pea genomes^15^,^16^,^17^. Notably, the position of RPW8 genes of Chinese chestnut were located at a relatively basal, but not the most basal position on the phylogenetic tree.

### The *cis*-element analysis of NBS-encoding genes promoter sequences

Plant defense is controlled by *cis*-regulatory elements corresponding to key genes involved in defense, and pathogen-specific responses^42^. Therefore, the investigation and identification of *cis*-acting elements in the promoters of NBS-encoding genes will help us understand the function in plant defense responses. In this analysis, we performed the *cis-*elemtne analysis of all NBS-encoding genes in the PLACE. It is noted that many *cis*-regulatory elements associate with plants responding to pathogens, including DOFCOREZM, EECCRCAH1, GT1GAMSCAM4, GT1CONSENSUS, and AGCBOXNPGLB^43^,^44^ in promoter regions of NBS-encoding genes (Table 3 and Additional file 3), which might demonstrate that NBS-encoding genes involve in response to pathogen infections. Moreover, the results (Additional file 3) point a way to identify candidate genes that might be used for conferring disease resistance.

## Discussion

### A small number of TIR-NBS-encoding genes in Chinese chestnut

Surveys for NBS-encoding genes in the sequenced genomes of many species, including *Arabidopsis*, rice, grapevine, and poplar have found variable numbers of NBS-encoding genes^2^,^19^,^20^,^21^. Based on the structure of the N-terminal domain, NBS-encoding genes have been categorized into two classes, TIR type and non-TIR type. TNLs were absent in monocots, such as rice^19^,^20^, the dicot *Aquilegia coerulea*^14^, and the dicotyledonous order Lamiales, but were found in most dicots^10^,^45^,^46^. However, only 27 TIR-type genes including 22 TNL genes and five TN genes, were identified in Chinese chestnut. The proportion of TNLs in entire genome of Chinese chestnut was only 0.0577%, which was much lower than that in *Arabidopsis* (0.348%), grapevine (0.319%), or poplar (0.171%)^2^,^21^. Furthermore, the percentage of TNLs among the NBS-LRR genes of poplar (23.6%) was four times to that in Chinese chestnut (5.88%), which also demonstrated that the number of TNLs was lower in Chinese chestnut. In Chinese chestnut, the average number of exons estimated for TNLs (2.50) was lower than that in whole genome genes and non-TNLs, a result that differed from those of previous studies in *Arabidopsis,* poplar, grapevine, apple, pear, and peach^2^,^21^,^47^.

Furthermore, because of the small number of TIR-type NBS-encoding genes, only two *Ks* values (0.4781 and 0.1623) were estimated for the two TIR gene families. The average *Ka/Ks* ratio for TIR-type genes, with *Ks* ranging from 0 to 1, was lower than that for non-TIR-type genes (0.4080 compared with 0.5871). This result was consistent with those of previous studies of TNLs and non-TNLs in *Arabidopsis*, grapevine, poplar, apple, pear, and peach^2^,^21^,^47^. The lower *Ka/Ks* values for TIR gene families than for non-TIR gene families indicates greater functional constraints during the evolution of TIR gene subfamilies^48^ and stronger diversifying selection in non-TIR gene families, which could provide variation that would allow plants to adapt to different pathogens in their environment^47^.

### Duplication events of NBS-encoding genes in Chinese chestnut

Gene duplication plays a critical role in the generation of new *R* genes, increasing the number of these genes and dispersing them in the genome^7^. The number of NBS-encoding genes in Chinese chestnut was 3- and 1.2-fold that in *Arabidopsis* and poplar, respectively. To identify duplicated gene pairs, we defined a gene family according to three criteria. Using 70% criterion of both cutoff of coverage and sequence identity of not less than 70%, 64 gene families were detected, including 273 NBS-encoding genes in Chinese chestnut (53.60%) (Table 2). The percentage of NBS-encoding genes occurring in multi-gene families in Chinese chestnut was nearly the same as that in the rice genome (53.7%) and lower than that in the poplar genome (78.1%). Using our 80% criterion of both cutoff of coverage and sequence identity of not less than 80%, 41.43% of NBS genes were found to belong to multi-gene families in Chinese chestnut, while 74.52% of NBS genes occurred in multi-gene families in the poplar genome. However, the proportion of genes in multi-gene families sharply declined to 19.46% when the 90% criterion was used, which showed that relatively recent duplications resulted in a small portion of the multi-gene families in Chinese chestnut.

The number of synonymous substitutions per synonymous site and the frequency distribution of *Ks* values could be used to infer the age of genome duplications^21^. The peak and distribution of *Ks* values was distinct in different species. *Ks* peaks varied greatly, occurring at Ks values from 0 to 0.1 in grape and poplar, and at Ks values from 0.1 to 0.2 in *Arabidopsis*, apple, pear, peach, and *Prunus mume*, but at *Ks* values from 0.3 to 0.4 in rice and strawberry^21^,^47^. In Chinese chestnut, *Ks* peaked in the range of values from 0.4 to 0.5 (Fig. 1), a much higher *Ks* value than that in poplar (*Ks* = 0–0.1) or in other species, indicating that the gene expansions of Chinese chestnut were more ancient. Furthermore, the *Ks* values for Chinese chestnut were mainly distributed in a range from 0.2 to 0.5 and were rarely greater than 0.5 (Fig. 1). The fact that 63.94% of multiple NBS-encoding genes were present in the range of *Ks* values from 0.2 to 0.5, demonstrating that relatively ancient duplications played an important role in the expansion of NBS-encoding genes in Chinese chestnut.

### Species-specific duplication driving in the expansion of NBS-encoding genes in Chinese chestnut

**Figure 3:**
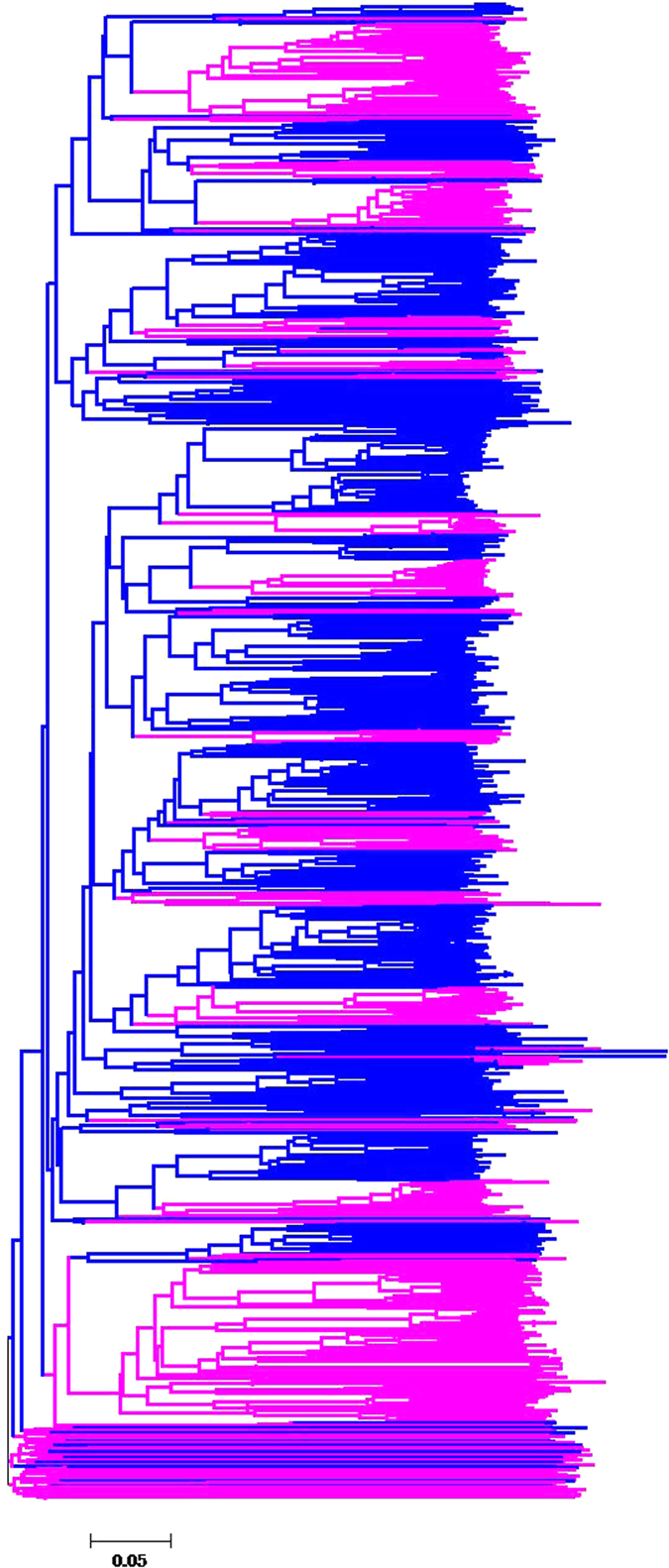
Phylogenetic tree of NBS domains from NBS-encoding genes. Blue (Chinese chestnut), purple (poplar).

Gene duplication has supplied raw genetic material for evolution^49^ and has been a major force for generating biological novelties that can lead to adaptation to environments^50^. To elucidate the evolutionary pattern of NBS-encoding genes in Chinese chestnut, a phylogenetic tree of conserved NBS domain from Chinese chestnut and poplar genomes was constructed (Fig. 3). The most distinct characteristic was species-specific duplication events, that might be responsible for the evolution of recognition of species-specific pathogens, and that responsed to selective pressure imposed by species-specific pathogens^21^.

The 401 species-specific NBS-encoding genes detected in Chinese chestnut were classified into 81 clades with bootstrap values were greater than 50%. The proportion of the species-specific duplicates reached 77.26%, suggesting large-scale genes expansion after the divergence of Chinese chestnut and poplar. A similar result has also been reported for four gramineous plants (*Zea mays, Sorghum bicolor, Brachypodium distachyon*, and *Oryza sativa*)^51^, and five species in the Rosaceae (*Fragaria vesca, Malus* *×* *domestica, Pyrus bretschneideri, Prunus persica, and Prunus mume*)^47^.

### The independent and relatively basal NBS group containing the RPW8 domain

The RPW8 domain (PF05659.6) mediated broad-spectrum pathogen resistance and was originally identified in the protein encoded by the polymorphic powdery mildew resistance locus RPW8^12^. In previous studies, proteins containing the RPW8 domain have been categorized into the non-TIR-NBS-encoding genes subgroup^13^,^14^. However, 15 RPW8 domain-encoding genes (1 XNs, 2 TNLs, 3 CNLs and 9 XNLs) clustered into an independent lineage in phylogenetic relationships (Additional file 2: Figure S1). Also, five NBS-encoding-genes from poplar encoding the RPW8 domain clustered together with 15 RPW8-NBS-encoding genes from Chinese chestnut. The clustering of RPW8-NBS-encoding genes observed in Chinese chestnut also occurred with RPW8-NBS-LRR genes from *M. truncatula*^15^, potato^16^, soybean, common bean, and pigeon pea^17^, which illustrated that RPW8-NBS-encoding genes did not comprise a special group of non-TIR genes but formed a separate group independent from the non-TIR genes. Moreover, the group of RPW8-NBS-encoding genes located at a relatively basal but not the most basal position in this phylogenetic tree of Chinese chestnut (Additional file 2: Figure S1). This result is similar to the study in *Arabidopsis* that a monophyletic group (the CNL-A group) consisted of RNL genes^2^,^17^. Thus, the RPW8-NBS-encoding genes were phylogenetically separate from the non-TIR genes.

## Materials and Methods

### Identification of NBS-encoding genes in Chinese chestnut and classification of gene family members

The entire genome sequence and annotation V1.0 of Chinese chestnut (*C. mollissima*) were downloaded from the hardwood genomics project (http://www.hardwoodgenomics.org/chinese-chestnut-genome). To identify NBS-encoding genes in Chinese chestnut, we used the amino acid sequence of the NB-ARC domain (PF00931) as blastp query against all known protein sequences with the threshold expectation value set to 1.0. All hits were further submitted to Pfam analysis (http://pfam.xfam.org/) to verify the presence of the NBS (NB-ARC) domain. Furthermore, the identified NBS-encoding genes were examined to detect whether they encode the LRR or TIR domain using the merged results from Pfam analysis and SMART protein motif analysis (http://smart.embl-heidelberg.de/). Finally, all identified genes were examined to detect the presence of CC domain using COILS (http://embnet.vital-it.ch/software/COILS_form.html) databases with a threshold of 0.9^52^ in Chinese chestnut and RPW8 domain using Pfam analysis in Chinese chestnut and poplar.

Genes were grouped into gene families according to two criteria, including the cutoff of coverage (aligned sequence lengths/gene lengths), and the sequence identity of not less than 70%. Likewise, the stricter criteria for both coverage and sequence identity of not less than 80% and 90%, respectively, were used to detect the relatively recent duplications among NBS-encoding genes.

### Sequence alignment and phylogenetic analyses

The NBS domain sequences of all identified NBS-encoding genes were aligned in MEGA 5.0 using the MUSCLE program^53^. A neighbor-joining (NJ) method was then applied to construct a phylogeny of NBS-encoding genes using ClustalW 2.0 with default options and 1000 bootstrap replications^54^. Data for NBS-encoding genes from poplar (*Populus trichocarpa*) were obtained from a previous study^21^.

### The ratio of nonsynonymous substitutions to synonymous substitutions

To detect the mode of selection, we evaluated the ratio of nonsynonymous substitutions to synonymous substitutions. Firstly, based on the protein sequences, the CDSs (nucleotide coding sequences) of NBS-encoding genes in each gene family were aligned using ClustalW 2.0^54^. Subsequently, nonsynonymous substitutions (*Ka*), synonymous substitutions (*Ks*), and the ratio between them (*Ka/Ks*) were calculated in each gene family using MEGA 5.0^53^.

### Detection of positive selection

The Phylogenetic Analysis by Maximum Likelihood 4 (PAML4) package^55^ was used for the site model and branch model test to determine selective pressure on NBS-LRR genes in Chinese chestnut. A single *dN/dS* ratio (model = 0), in addition to models M7 (beta) and M8 (beta-ω) (NS site = 7 8) were used for the site model in all gene families with at least three members. Subsequently, the LR test between model M7 and M8 was carried out with critical criteria of chi-square 5.991 (*p* < 0.05, *df* = 2) and 9.210 (*p* < 0.01, *df* = 2), respectively. For the branch model, a single *dN/dS* ratio (model = 0) and model 0 (NS site = 0) were applied in the codeml program.

### Promoter regions analysis

To characterize the *cis*-acting element(s) of NBS-encoding genes, we isolated an approximately 1000 bp promoter sequence of each NBS-encoding gene. Analysis of promoter sequences was conducted using SIGNALSCAN program available in Plant cis-acting regulatory DNA Elements (PLACE) (http://www.dna.affrc.go.jp/PLACE/), a database containing mainly plant motifs extracted from the published reports^56^.

## Conclusions

NBS-LRR genes, as one of the largest families of *R* genes, were analyzed in Chinese chestnut (*Castanea mollissima*), a model species for the Fagaceae, to determine their pattern of evolution. In the present study, several TNLs were identified in Chinese chestnut that were absent from monocot genomes. In addition, we found a relatively ancient duplication in Chinese chestnut compared with poplar. The expansion of NBS-encoding genes could be attributed to such species-specific duplications during the evolution of Chinese chestnut. The values for *Ka/Ks* in all TIR and most non-TIR gene families were less than 1, indicating purifying selection as a leading force in the evolution of NBS-encoding genes. However, the *Ka/Ks* values for four non-TIR gene families were greater than 1, demonstrating that their evolution was driven by positive selection. Furthermore, the relationship between *Ka/Ks* and *Ks* illustrated higher selective pressure on the newer and older genes compared with genes in the critical range of *Ks* from 0.4 to 0.5. Interestingly, RPW8-NBS-encoding genes clustered into an independent clade at a relatively basal, but not most basal, position in this phylogenetic analysis. Finally, many *cis*-elements in NBS-encoding genes promoter were related to disease resistance, which demonstrated the function in responsing pathogens and laid the foundation of identifying candidate *R* genes.

## Additional Information

**How to cite this article**: Zhong, Y. *et al.* Species-specific duplications of NBS-encoding genes in Chinese chestnut (*Castanea mollissima*). *Sci. Rep.* **5**, 16638; doi: 10.1038/srep16638 (2015).

## Supplementary Material

Supplementary Information

Supplementary Information

## Figures and Tables

**Table 1:** The number of NBS-encoding genes in the Chinese chestnut genome.

Predicted protein domains	Letter code	Castanea mollissima	Populus trichocarpa^a^
**NBS-encoding genes**		519	416
**NBS-LRR type**		374	330
TIR-NBS-LRR	TNL	22	78
non-TIR-NBS-LRR	non-TNL	352	252
CC-NBS-LRR	CNL	32	120
X-NBS-LRR	XNL	320	132
** NBS**		145	86
TIR-NBS	TN	5	10
non-TIR-NBS	non-TN	140	76
CC-NBS	CN	96	14
X-NBS	XN	44	62
genes from the entire genome		38081	45555
Proportion of NBS-encoding genes		1.36%	0.91%
Proportion of NBS-LRR genes		0.98%	0.72%
Proportion of TIR-NBS-LRR genes		0.06%	0.17%
Proportion of non-TIR-NBS-LRR genes		0.92%	0.55%
Average exon of all genes		4.37	2.35
Average exon of TIR-NBS-LRR		2.50	3.5
Average exon of non-TIR-NBS-LRR		2.93	2.23
Average exon of CC-NBS-LRR		3.32	–b
Average exon of NBS-encoding genes		2.66	–
Average exon of NBS-LRR genes		2.90	–

^a^Data from Yang *et al*. (2008).

^b^Not given in Yang *et al.* (2008).

**Table 2:** Classification of NBS-encoding genes from Chinese chestnut.

Gene family	C. mollissima			P. trichocarpa	
	70%	80%	90%	70%	80%
Multi-gene	273	215	101	325	310
Single gene	246	304	418	91	106
Proportion of multiple genes	52.60%	41.43%	19.46%	78.13%	74.52%
Gene Family No.	64	61	41	61	64
Average number of members/family	4.27	3.52	2.46	5.33	4.84
Maximal members of a family	19	17	5	23	17
TIR multiple genes	4	2	0	–a	–
TIR multi-gene family No.	2	1	0	–	–
Proportion of TIR multiple genes	14.81%	7.41%	0.00%	–	–
non-TIR multiple genes	269	213	101	–	–
non-TIR multi-gene family No.	62	60	41	–	–
Proportion of non-TIR multiple genes	54.67%	43.29%	20.53%	–	–

^a^Not given in Yang *et al*. (2008).

**Table 3:** The *cis*-acting element analysis of NBS-encoding genes promoter sequences.

Element name	Number	Element name	Number	Element name	Number	Element name	Number
DOFCOREZM	495	CAREOSREP1	172	MYBATRD22	47	GBOXLERBCS	4
CACTFTPPCA1	487	IBOX	172	GARE1OSREP1	46	HEXAT	4
CAATBOX1	486	TATABOX3	172	DRE2COREZMRAB17	44	MNF1ZMPPC1	4
GT1CONSENSUS	482	SORLIP2AT	170	REBETALGLHCB21	42	OPAQUE2ZMB32	4
GTGANTG10	481	MYBGAHV	169	ACGTABOX	41	RGATAOS	4
ARR1AT	477	SREATMSD	167	HDZIP2ATATHB2	40	S2FSORPL21	4
GATABOX	475	ERELEE4	163	SURE1STPAT21	36	SORLIP4AT	4
POLLEN1LELAT52	475	WBOXNTCHN48	162	PALBOXAPC	35	SORLREP4AT	4
WRKY71OS	472	WBBOXPCWRKY1	160	TRANSINITDICOTS	35	TELOBOXATEEF1AA1	4
ROOTMOTIFTAPOX1	454	SEF1MOTIF	149	SORLREP3AT	34	UPRMOTIFIAT	4
NODCON2GM	452	SP8BFIBSP8BIB	149	ACGTABREMOTIFA2OSEM	33	ANAERO5CONSENSUS	3
OSE2ROOTNODULE	452	ASF1MOTIFCAMV	148	BOXCPSAS1	33	HBOXCONSENSUSPVCHS	3
EBOXBNNAPA	451	ELRECOREPCRP1	145	QARBNEXTA	33	HSELIKENTACIDICPR1	3
MYCCONSENSUSAT	451	SEBFCONSSTPR10A	144	CARGATCONSENSUS	32	MRNA3ENDTAH3	3
TAAAGSTKST1	446	GT1CORE	136	ATHB5ATCORE	29	NONAMERATH4	3
WBOXNTERF3	432	RYREPEATBNNAPA	136	UP1ATMSD	27	OCTAMERMOTIFTAH3H4	3
TATABOX5	431	LECPLEACS2	130	EVENINGAT	25	PALBOXPPC	3
GT1GMSCAM4	428	RBCSCONSENSUS	126	GCCCORE	25	PIATGAPB	3
POLASIG1	428	ARFAT	124	IRO2OS	23	SP8BFIBSP8AIB	3
CCAATBOX1	423	MYBPLANT	122	NRRBNEXTA	23	SPHCOREZMC1	3
INRNTPSADB	422	T/GBOXATPIN2	122	PROXBBNNAPA	23	ABREATRD22	2
SEF4MOTIFGM7S	422	PYRIMIDINEBOXHVEPB1	119	S1FSORPL21	22	ABREMOTIFAOSOSEM	2
RAV1AAT	417	PRECONSCRHSP70A	118	SURE2STPAT21	22	BOX2PSGS2	2
IBOXCORE	414	TATAPVTRNALEU	118	BOXIIPCCHS	21	CAATBOX2	2
POLASIG3	404	CBFHV	115	ATHB1ATCONSENSUS	20	E2F1OSPCNA	2
WBOXATNPR1	392	MYB2AT	115	CMSRE1IBSPOA	20	E2FANTRNR	2
WBOXHVISO1	379	SV40COREENHAN	115	AGMOTIFNTMYB2	19	ELRENTCHN50	2
BIHD1OS	375	BOXLCOREDCPAL	114	BP5OSWX	19	GMHDLGMVSPB	2
MYBCORE	367	IBOXCORENT	113	GARE2OSREP1	19	JASE1ATOPR1	2
CURECORECR	364	AACACOREOSGLUB1	111	ANAERO4CONSENSUS	17	LBOXLERBCS	2
EECCRCAH1	362	2SSEEDPROTBANAPA	110	LTREATLTI78	17	LREBOXIIPCCHS1	2
MARTBOX	352	CATATGGMSAUR	108	HEXAMERATH4	16	PALINDROMICCBOXGM	2
ACGTATERD1	339	CCA1ATLHCB1	105	ABREOSRAB21	15	PE2FNTRNR1A	2
MYB1AT	336	E2FCONSENSUS	105	ACGTOSGLUB1	15	ABRE3HVA1	1
NODCON1GM	333	QELEMENTZMZM13	105	ATHB6COREAT	15	ABREAZMRAB28	1
OSE1ROOTNODULE	333	LTRECOREATCOR15	101	AUXREPSIAA4	15	ACGTABREMOTIFAOSOSEM	1
MYBST1	327	CGCGBOXAT	100	BS1EGCCR	15	ACGTROOT1	1
-300ELEMENT	314	TATCCAYMOTIFOSRAMY3D	100	ABREATCONSENSUS	13	ACIPVPAL2	1
NTBBF1ARROLB	313	-300CORE	93	DRE1COREZMRAB17	13	AT1BOX	1
REALPHALGLHCB21	312	ANAERO2CONSENSUS	92	MYB26PS	13	AUXRETGA2GMGH3	1
POLASIG2	300	LTRE1HVBLT49	91	SBOXATRBCS	12	BOX1PSGS2	1
DPBFCOREDCDC3	290	RYREPEATLEGUMINBOX	85	UP2ATMSD	12	C2GMAUX28	1
PYRIMIDINEBOXOSRAMY1A	285	PROLAMINBOXOSGLUB1	83	AMMORESIVDCRNIA1	11	CONSERVED11NTZMATP1	1
SURECOREATSULTR11	276	WUSATAg	80	AGCBOXNPGLB	10	CPRFPCCHS	1
-10PEHVPSBD	268	MARABOX1	79	ATHB2ATCONSENSUS	10	D1GMAUX28	1
ANAERO1CONSENSUS	268	CTRMCAMV35S	72	ACGTCBOX	9	DE1PSPRA2	1
BOXIINTPATPB	268	MYB1LEPR	72	AMMORESIIUDCRNIA1	9	DR5GMGH3	1
TBOXATGAPB	268	XYLAT	68	CRTDREHVCBF2	9	E2FBNTRNR	1
MYB2CONSENSUSAT	256	MARARS	66	UPRMOTIFIIAT	9	GRAZMRAB28	1
CARGCW8GAT	243	DRECRTCOREAT	65	CARGNCAT	8	GT2OSPHYA	1
ABRELATERD1	238	INTRONLOWER	65	INTRONUPPER	8	HDMOTIFPCPR2	1
CIACADIANLELHC	236	P1BS	63	LRENPCABE	8	HSRENTHSR203J	1
SEF3MOTIFGM	231	ACGTTBOX	62	ZDNAFORMINGATCAB1	8	L1DCPAL1	1
SORLIP1AT	230	GT1MOTIFPSRBCS	62	EMBP1TAEM	7	LREBOXIPCCHS1	1
CPBCSPOR	226	L1BOXATPDF1	62	GGTCCCATGMSAUR	7	NONAMERMOTIFTAH3H4	1
TATABOX2	220	ANAERO3CONSENSUS	61	GLMHVCHORD	7	O2F1BE2S1	1
PREATPRODH	210	RAV1BAT	61	RYREPEATVFLEB4	7	OPAQUE2ZM22Z	1
TATABOX4	206	CGACGOSAMY3	59	-300MOTIFZMZEIN	6	RBCSBOX3PS	1
GAREAT	205	GCN4OSGLUB1	58	ABREZMRAB28	6	RBCSGBOXPS	1
RHERPATEXPA7	203	SORLIP5AT	58	AGL2ATCONSENSUS	6	SGBFGMGMAUX28	1
TATABOXOSPAL	203	CEREGLUBOX2PSLEGA	57	D4GMAUX28	6	SORLREP2AT	1
AMYBOX1	201	NAPINMOTIFBN	56	POLLEN2LELAT52	6	SORLREP5AT	1
CANBNNAPA	197	AMYBOX2	55	SITEIOSPCNA	6	TATABOX1	1
S1FBOXSORPS1L21	191	TRANSINITMONOCOTS	55	AGATCONSENSUS	5	TE2F2NTPCNA	1
SITEIIATCYTC	184	CACGTGMOTIF	54	AUXRETGA1GMGH3	5	TOPOISOM	1
MYCATERD1	182	RYREPEATGMGY2	54	CACGCAATGMGH3	5	VOZATVPP	1
MYCATRD22	182	ARE1	53	PALBOXLPC	5	VSF1PVGRP18	1
MYBPZM	181	HEXMOTIFTAH3H4	51	SORLIP3AT	5	WRECSAA01	1
MYBCOREATCYCB1	180	TATCCACHVAL21	50	5659BOXLELAT5659	4		
TATCCAOSAMY	174	LEAFYATAG	49	ACIIIPVPAL2	4		
ABRERATCAL	172	EMHVCHORD	47	DREDR1ATRD29AB	4		
