# Supplementary material for: Species-specific duplications of NBS-encoding genes in Chinese chestnut (Castanea mollissima)
Source: Sci Rep. 2015 Nov 12;5:16638. doi: 10.1038/srep16638 (PMC4642323; doi:10.1038/srep16638)

SREP-15-19187A:

Species-specific duplications of NBS-encoding genes in Chinese chestnut (*Castanea mollissima*)

Yingjun Li[1] , Kaihui Huang[1] , Zong-Ming Cheng[1] & Yan Zhong[1, *]

[1] College of Horticulture, Nanjing Agricultural University, Nanjing 210095, China

[*]Corresponding author (E-mail: yzhong@njau.edu.cn; Telephone, 86-25- 84396055)

Additional files

Additional file 1

Table S1. Positive selection sites in NBS-encoding genes in Chinese chestnut.
* and ** represent the sites under positive selection at the cutoffs Pb = 95% and 99%, respectively.

Additional file 2

Figure S1. Species-specific clades of Chinese chestnut and the distribution of RPW8 domain-containing NBS-encoding genes in Chinese chestnut and poplar in phylogenetic tree. Vertical red lines represent the clades; black dots and red triangles represent NBS-encoding genes containing an RPW8 domain in Chinese chestnut and poplar, respectively.

Additional file 3 (.zip)

The result of *cis*-element analysis of NBS-encoding genes promoter sequences.

Table S1. Positive selection sites of NBS-encoding genes in Chinese chestnut

AA site		AA site		AA site	
**family0**		291 S	**	367 E	**
138 P	*	312 S	**	390 A	*
171 G	*	396 S	*	393 N	*
435 F	**	**family15**		413 S	*
478 Y	**	1031 S	*	447 L	**
479 T	*	family17		448 T	**
523 S	**	50 A	**	449 L	**
546 G	*	51 N	**	450 N	**
547 S	*	212 S	*	454 F	**
569 D	**	**family18**		455 Y	*

570 F	**	148 S	*	459 V	*
594 L	*	150 V	**	519 F	*
595 E	*	152 R	*	537 I	*
625 H	**	196 D	*	576 Y	*
651 H	*	200 Q	**	601 Q	**
656 Q	*	295 A	**	621 D	**
671 D	*	345 F	**	623 E	*
718 Y	**	347 R	**	625 F	**
751 E	*	349 D	**	626 D	**
776 S	**	369 Q	*	641 N	*
777 S	*	391 E	*	655 V	**
806 V	*	418 C	**	675 R	*
835 G	*	442 R	**	677 L	**
836 L	**	**family23**		697 E	*
859 S	**	1058 Y	*	700 D	*
860 E	*	**family24**		718 R	*
907 R	*	40 S	**	720 S	*
**family1**		44 R	*	745 G	**
448 N	**	90 L	*	770 L	*
**family2**		106 C	*	773 R	*
162 H	*	114 Y	*	775 R	*
family4		137 N	*	**family41**	
21 R	**	232 T	*	97 M	*
32 L	*	243 D	*	102 M	*
34 N	*	247 V	*	105 Y	*
37 T	*	261 G	*	106 F	*
49 R	*	309 G	*	107 D	*
73 V	*	329 G	*	110 V	*
77 I	**	371 L	*	111 L	*
102 I	*	380 F	*	118 D	**
108 Q	*	480 I	*	119 I	**
117 P	*	485 K	*	120 Y	**
204 K	*	506 K	*	122 Y	**
429 K	**	509 P	*	123 K	**
641 L	*	512 R	*	125 H	**
**family6**		524 P	*	130 D	*
10 I	*	535 F	**	131 S	*
342 A	*	561 A	*	132 E	*
372 D	*	563 K	*	133 R	*
414 E	*	566 S	**	137 R	*
420 F	*	586 E	*	144 H	*
468 I	*	610 K	*	145 L	*
493 S	*	778 L	*	147 G	*
514 L	*	784 R	*	148 S	*

516 A	*
519 A	*
520 K	*
534 Y	**
543 E	*
551 A	*
560 Y	*
561 W	*
563 Q	**
565 T	*
574 S	*
576 G	*
580 R	*
586 S	**
592 S	**
603 P	*
609 Q	**
623 Y	**
624 N	*
646 D	*
650 L	*
651 G	*
653 R	*
662 S	**
663 Q	**
667 F	*
669 Y	*
670 M	**
**family9**	
39 L	**
42 A	**
95 Y	*
99 S	*
135 L	**
182 R	**
216 T	**
235 P	**
244 Q	**
297 H	**
301 F	*
348 F	*
351 E	**
393 D	**
394 L	*
787 T	*
906 E	*
910 V	*
940 S	*
955 M	*
963 G	**
1014 G	**
1039 L	*
1049 W	*
**family28**	
105 Y	*
179 L	*
254 V	*
319 I	**
348 I	*
365 S	*
378 P	**
396 T	*
546 D	**
549 Q	**
551 S	**
552 Y	**
572 L	*
579 R	**
645 A	**
647 Y	**
681 D	*
683 D	**
736 N	**
779 R	**
805 R	**
807 Y	**
808 N	**
829 L	*
832 R	**
853 E	*
856 E	**
880 S	**
882 Y	**
884 V	**
885 N	*
**family29**	
191 S	*
412 S	*
150 E	*
153 G	*
155 G	*
157 L	*
252 R	*
299 Q	*
**family46**	
24 N	*
31 R	*
34 G	*
106 Q	*
118 S	*
207 K	*
209 V	*
232 K	*
382 K	*
383 Q	*
395 L	*
425 Y	**
427 R	*
449 D	**
495 R	**
499 C	*
595 E	*
599 H	*
**family56**	
513 R	*
529 L	*
565 F	**
638 C	**
662 V	*
684 E	*
697 D	**
**family57**	
55 H	*
59 K	**
143 I	*
144 L	**
167 D	*
171 N	*
235 E	*
363 E	*
407 P	*
410 S	*

395 Y	**	640 G	*	427 V	*
415 H	**	641 F	*	435 N	**
434 L	*	726 V	*	436 I	**
436 N	**	763 C	*	441 A	*
438 I	**	933 Q	**	442 A	*
448 C	*	965 S	*	464 A	**
456 L	**	966 S	*	508 L	*
473 V	**	**family35**		521 K	*
474 T	*	88 I	**	532 R	**
494 H	**	135 E	*	534 E	**
496 E	**	167 N	*	583 K	*
498 E	**	169 F	**	608 R	*
501 Y	*	189 L	**	646 R	*
520 I	**	271 V	*	667 N	**
522 I	**	275 N	**	669 F	**
523 D	**	398 R	**	670 N	**
541 I	**	441 K	*	689 R	*
543 R	**	467 S	**	738 R	*
544 D	**	470 G	**	761 L	*
**family10**		492 S	**	763 E	**
1115 C	*	494 D	**	764 H	*
1231 P	**	516 L	**	784 N	**
**family11**		519 T	*	818 R	*
557 N	*	540 G	**	839 N	**
565 C	**	542 V	**	840 L	*
673 K	*	543 Y	*	842 S	*
674 V	**	564 E	**		
**family12**		595 Y	**		
4 L	**	597 Q	**		
81 E	*	599 S	**		
99 Q	*	601 S	*		
125 A	*	**family39**			
193 D	*	11 A	*		
266 E	*	155 G	*		
267 S	**	163 H	*		
269 F	**	317 G	*		

*** and ** represent the sites under positive selection at the cutoffs P_b = 95% and 99%.**

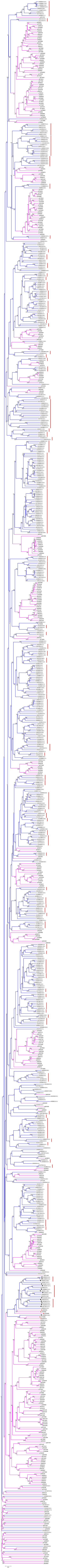

Supplement: Supplementary Information [file srep16638-s1.pdf]
